# Research Trends in Artificial Intelligence Applications in Human Factors Health Care: Mapping Review

**DOI:** 10.2196/28236

**Published:** 2021-06-18

**Authors:** Onur Asan, Avishek Choudhury

**Affiliations:** 1 School of Systems and Enterprises Stevens Institute of Technology Hoboken, NJ United States

**Keywords:** artificial intelligence, human factors, health care systems, ecological validity, usability, trust, perception, workload

## Abstract

**Background:**

Despite advancements in artificial intelligence (AI) to develop prediction and classification models, little research has been devoted to real-world translations with a user-centered design approach. AI development studies in the health care context have often ignored two critical factors of ecological validity and human cognition, creating challenges at the interface with clinicians and the clinical environment.

**Objective:**

The aim of this literature review was to investigate the contributions made by major human factors communities in health care AI applications. This review also discusses emerging research gaps, and provides future research directions to facilitate a safer and user-centered integration of AI into the clinical workflow.

**Methods:**

We performed an extensive mapping review to capture all relevant articles published within the last 10 years in the major human factors journals and conference proceedings listed in the “Human Factors and Ergonomics” category of the Scopus Master List. In each published volume, we searched for studies reporting qualitative or quantitative findings in the context of AI in health care. Studies are discussed based on the key principles such as evaluating workload, usability, trust in technology, perception, and user-centered design.

**Results:**

Forty-eight articles were included in the final review. Most of the studies emphasized user perception, the usability of AI-based devices or technologies, cognitive workload, and user’s trust in AI. The review revealed a nascent but growing body of literature focusing on augmenting health care AI; however, little effort has been made to ensure ecological validity with user-centered design approaches. Moreover, few studies (n=5 against clinical/baseline standards, n=5 against clinicians) compared their AI models against a standard measure.

**Conclusions:**

Human factors researchers should actively be part of efforts in AI design and implementation, as well as dynamic assessments of AI systems’ effects on interaction, workflow, and patient outcomes. An AI system is part of a greater sociotechnical system. Investigators with human factors and ergonomics expertise are essential when defining the dynamic interaction of AI within each element, process, and result of the work system.

## Introduction

### Influx of Artificial Intelligence in Health Care

The influx of artificial intelligence (AI) has been shifting paradigms for the last decade. The term “AI” has been often used and interpreted with different meanings [1], and there is a lack of consensus regarding AI’s definition [2]. In general, AI can be defined as a computer program or intelligent system capable of mimicking human cognitive function [3]. Over the years, the capabilities and scope of AI have substantially increased. AI now ranges from algorithms that operate with predefined rules and those that rely on if-then statements (decision tree classifiers) [4] to more sophisticated deep-learning algorithms that have the capabilities to automatically learn and improve through statistical analyses of large datasets [5,6]. There have been many studies and advancements with AI as it continues to evolve in numerous domains, including health care. AI applications such as MelaFind, a virtual assistant software, and IBM Watson have been introduced to improve health care systems, foster patient care, and augment patient safety [7]. AI applications have been developed and studied for every stakeholder in health care, including providers, administrators, patients, families, and insurers. In some specific areas such as radiology and pathology, there are strong arguments that AI systems may supersede doctors as a result of studies showing that AI algorithms outperformed doctors in accurately detecting cancer cells [8-10].

Further, developments in AI-enabled health information technologies (eg, AI-enabled electronic health records [EHRs] or clinical decision support systems) have benefitted from the availability of big data to predict clinical outcomes and assist providers in parsing through their EHRs to find individual pieces of medical information [11]. Despite AI having great potential, it is still in its infancy. The existing clinical AI systems are far from perfect for several well-known reasons, including (a) discriminatory biases coming from the input data; (b) lack of transparency in AI decisions, particularly neural networks, due to the black-box nature; and (c) sensitivity of the resulting decisions to the input data [6,12].

### Typical AI-User Interactions

**Figure 1:**
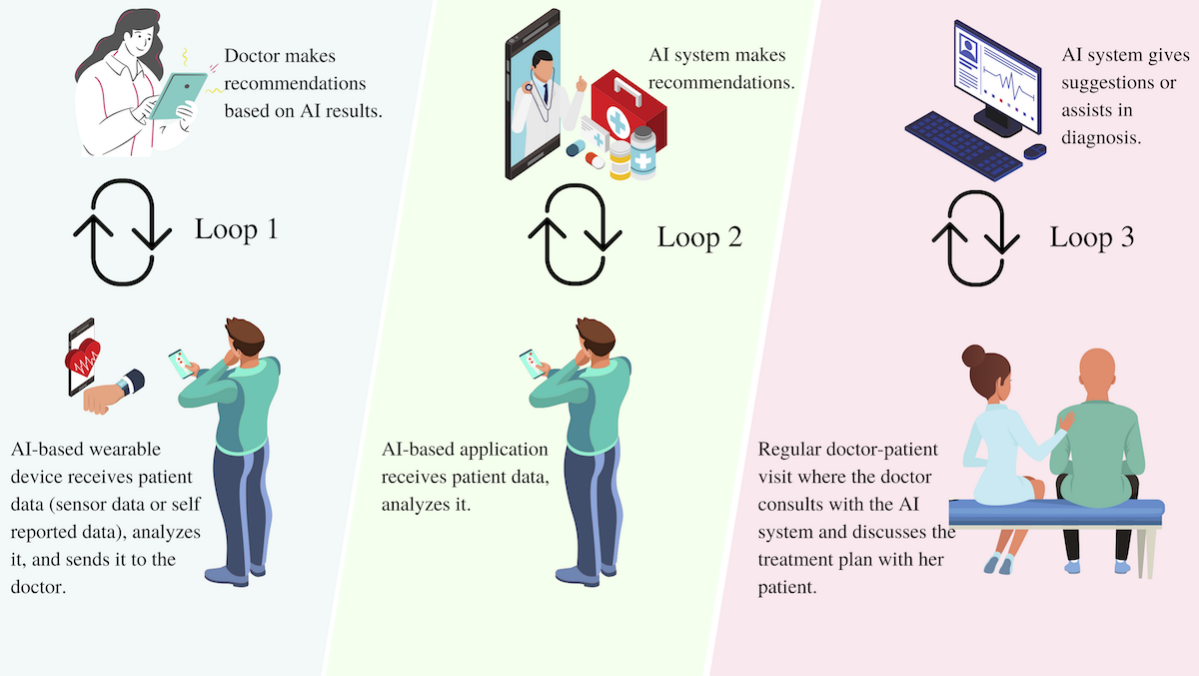
User-artificial intelligence (AI) interaction loops.

AI systems are complex in the sense of being a black box for the users who might not have adequate expertise in statistics or computer science to be able to comprehend the functioning of AI. Thus, AI can undesirably complicate the relationships between users and computer systems if not well designed. Unlike other health care technologies, AI can interact (eg, through chatbots, automated recommender systems, health apps) with clinicians and patients based on the inputs (feedback) that it receives from the user, thus creating what we refer to as “the interaction loop.” Unlike non-AI technologies, AI’s output (result generated by the AI) largely depends on the information fed into it; for instance, in AI-based reinforcement learning [13], the system may learn and adapt itself based on user input. Therefore, the human-AI interaction may influence the human as well as the AI system: the user feeds AI with some information; the AI learns from this information, performs analyses, and sends an output to the user; the user receives the output, comprehends it, and acts accordingly; and the new data generated by the user’s action goes back to the AI. Figure 1 illustrates three fundamental and typical interaction loops highlighting fundamental plausible transactions among clinicians, patients, and the AI system, in which the AI technology (such as Apple Watch) continuously measures the user’s health information (heart rate, oxygen level) and sends the data to the user’s health care provider. The care provider can then make treatment plans or clinical recommendations based on the AI results, which will then influence user health or health-related behavior (Loop 1). Other common user-AI interactions can be observed in online health services in which the user interacts with an AI-enabled chatbot for preliminary diagnoses (Loop 2). The third, but less common, user-AI interaction is when a doctor and patient together leverage an AI system for obtaining a better diagnosis in a clinical environment (Loop 3). For all of these applications, it is essential for the users to make a correct interpretation of AI outcomes, and to have a basic understanding of AI requirements and limitations. The optimum and successful user-AI interaction depends on several factors, including the physical (eg, timely access to technology, and visual and hearing ability, particularly of patients), cognitive (eg, ability to comprehend AI functioning, ability to reason and use AI-enabled devices), and emotional (eg, current state of mind, willingness to use AI, prior experience with AI technology) resources of people (eg, health professionals and caregivers).

### Efforts to Improve AI and the Essential Role of Human Factors

**Figure 2:**
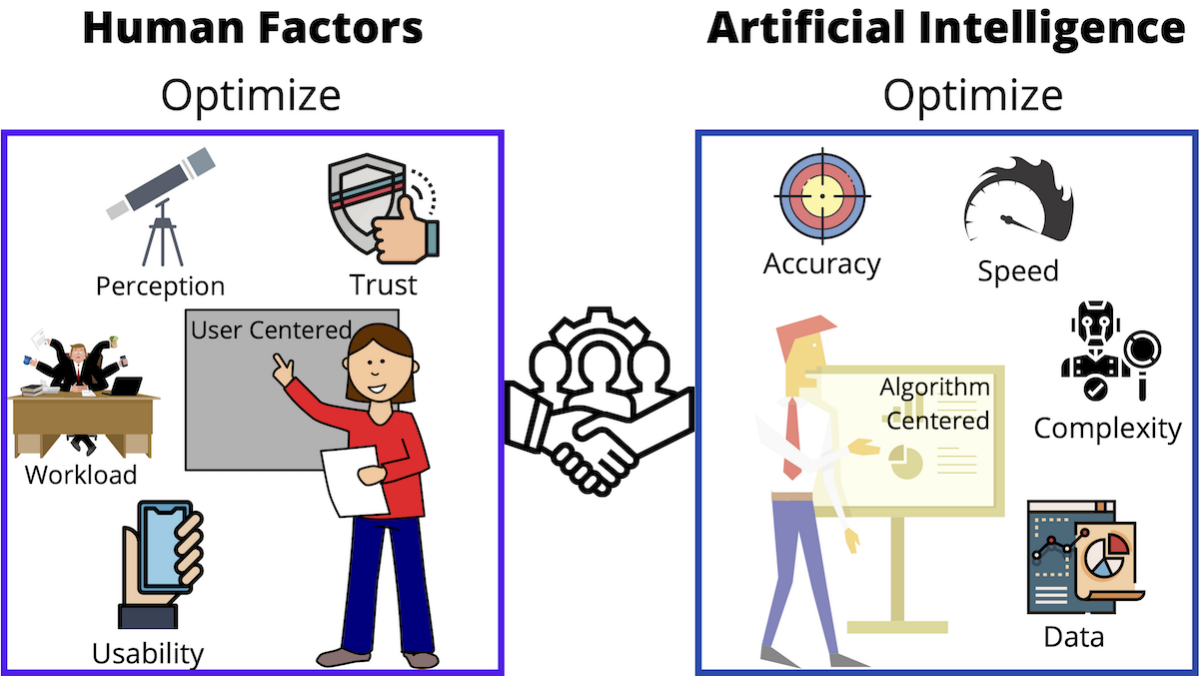
Illustrating some of the research objectives of experts in human factors and artificial intelligence.

The developers of health care AI apps have primarily focused on AI’s analytical capabilities, accuracy, speed, and data handling (see Figure 2) and have neglected human factors perspectives, which lead to poorly designed apps [14]. Although recent studies have reported the impact of biased data [15], as well as interpretability, interoperability, and lack of standardization [7,16] on AI outcomes, very few have acknowledged the need to assess the interactions among AI, clinicians, and care recipients.

Recently, as acknowledged in the Annual Meetings of the Human Factors Ergonomics Society [17,18], increasing autonomous activities in health care can pose risks and concerns regarding AI. Therefore, there is a need to integrate human factors and ergonomics (HFE) principles and methods into developing AI-enabled technologies for better use, workflow integration, and interaction. In health care AI research, two factors have not been sufficiently addressed by researchers, namely ecological validity and human cognition, which may create challenges at the interface with clinicians as well as the clinical environment and lead to errors. Moreover, there is insufficient research focusing on improving the human factors, mainly (a) how to ensure whether clinicians are implementing the AI correctly, (b) the cognitive workload it imposes on clinicians working in stressful environments, and (c) its impact on clinical decision-making and patient outcome. The inconvenient truth is that most of the AI showing prominent ability in research and the literature is not currently executable in a clinical environment [19,20]. Therefore, to better identify the current state of HFE involvement in health care AI, we performed a mapping review of studies published in major human factors journals and proceedings related to AI systems in health care. The aim of the mapping review was to highlight what has been accomplished and reported in HFE journals and discuss the roles of HFE in health care AI research in the near future, which can facilitate smoother human-system interactions.

## Methods

### Design and Data Source

We performed a mapping review to explore the trending and initial areas regarding health care AI research in HFE publications. Our protocol was registered with the Open Science Framework on October 2, 2020 [21]. Mapping reviews are well-developed approaches to cover the representative literature (not exhaustive) for exploring and demonstrating trends in a given topic and time duration [22]. In this study, we selected major human factors journals and conferences that potentially publish health care–related work as our data source. Our selection of journals and conferences was guided by the “Human Factors and Ergonomics” category of the Scopus Master List and Scimago Journal & Country Rank. We also added two journals that potentially publish patient safety-related human factors work: *Journal of Patient Safety* and *BMJ Quality and Safety*. In total, we explored 24 journals and 9 conference proceedings (see Multimedia Appendix 1). All the authors approved the final list of journals and conferences with consensus.

### Inclusion and Exclusion Criteria

We performed an extensive manual search to capture all relevant articles published in English within the last 10 years (January 2010 to December 2020) in the journals and conference proceedings listed in Multimedia Appendix 1. In each published volume, we searched for studies reporting qualitative or quantitative findings in the context of AI in health care. The selected studies needed to (1) be framed in the context of health care; (2) cover an AI algorithm or AI-enabled technology such as machine learning, natural language processing, or robotics; and (3) report either qualitative or quantitative findings/outcomes. We only included journal papers and full conference proceeding papers. Other materials such as conference abstracts, editorials, book chapters, poster presentations, perspectives, study protocols, review papers, and gray literature (eg, government reports and policy statement papers) were excluded.

### Paper Selection and Screening

Articles in the journal and conference list were manually screened by two reviewers (AC and a research assistant) based on titles and abstracts using one of the inclusion criteria (ie, to be framed in the context of health care). We exported all of the retrieved publications to Sysrev software. In the second step, we excluded all ineligible publications (eg, reviews, short abstracts, and posters), as explained in the preceding section. In the last step, two reviewers (AC and a research assistant) independently screened all of the selected full papers based on the remaining two inclusion criteria: (1) covering an AI algorithm or AI-enabled technology such as machine learning, natural language processing, or robotics; and (2) reporting either qualitative or quantitative findings/outcomes. The reviewers also confirmed that the studies were framed in a health care context. The reviewers achieved 82% agreement. The lead researcher (OA) then resolved all conflicts, screened all of the shortlisted full-text articles, and finalized the article selection.

### Data Extraction and Analysis

We followed a similar data extraction approach and analysis as reported by Holden et al [23]. Metadata (author names, the title of the paper, abstract) for each of the included articles were recorded in a standard Excel sheet. In our analysis, both authors (AC and OA) coded each included paper on different dimensions such as (1) sample/participant type, (2) AI system used, (3) source of data collection, and (4) objective and outcomes. Studies were also discussed based on the HFE principles such as evaluating workload, usability, trust in technology, perception, and user-centered design. These HFE principles and subcategories for the dimensions were derived from the final selected papers and were checked for face validity by the researchers. We iteratively worked on the data extraction process and revised the categories to achieve a final consensus.

## Results

### Summary of Included Studies

**Figure 3:**
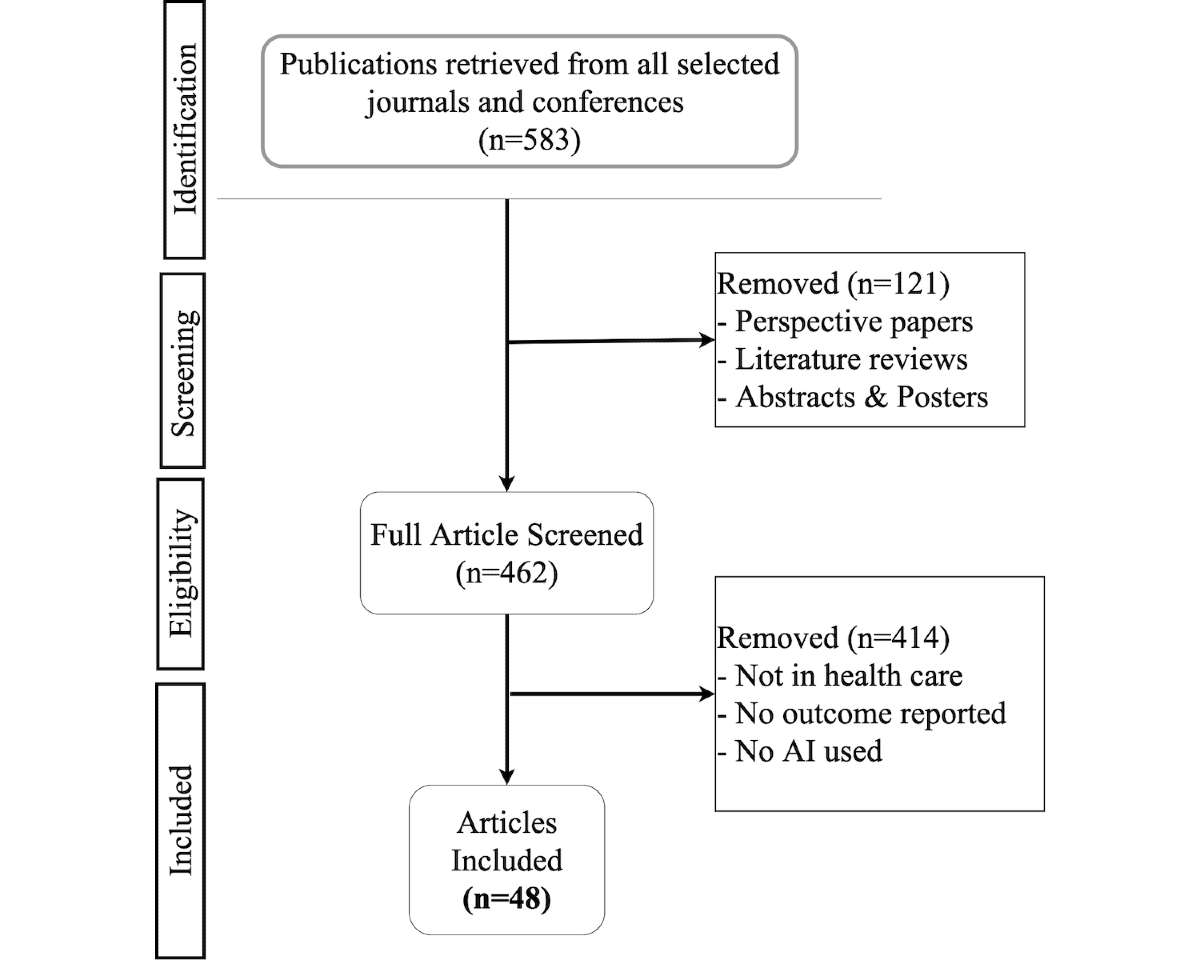
Selection and exclusion process. AI: artificial intelligence.

**Figure 4:**
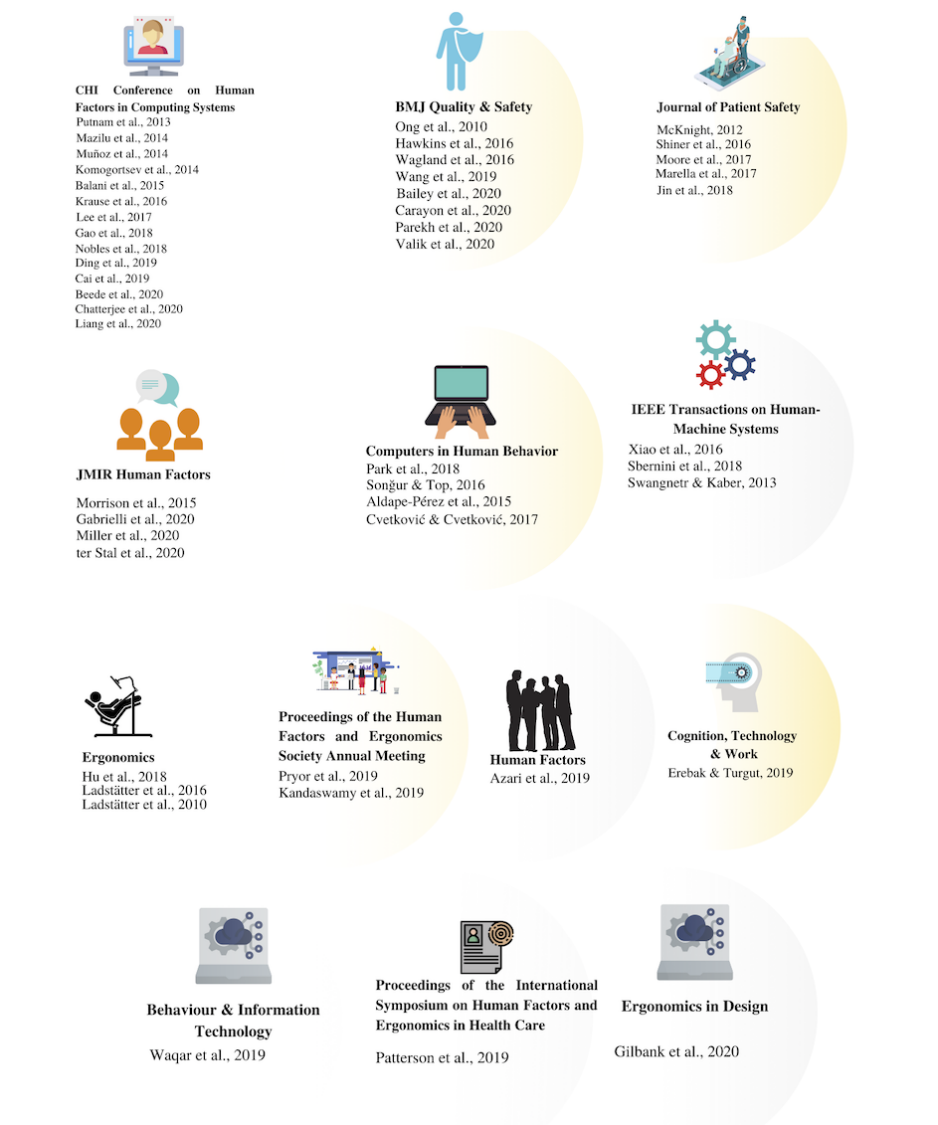
Overview of selected publications and their venues.

Figure 3 illustrates the screening and selection process. As a result of screening 24 selected journals and 9 conference proceedings (Multimedia Appendix 1), we finalized 48 articles matching our inclusion criteria, which were included in the scoping review with consensus from all reviewers. These 48 articles were published in 10 journals and 3 conference proceedings, as illustrated in Figure 4.

Table 1 shows the following dimensions: (1) objective of the study; (2) overall methods used, including the ethnographic/quantitative analysis methods adopted, and the type of data (“Methods and Data” column); (3) study participants (user of the AI system); and (4) primary outcome/findings of the study. Most studies involved human participants such as clinicians and patients (n=33) as shown in the “Study Participants” column in Table 1. However, some studies used data from online sources such as Reddit, Twitter, and clinical databases. Approximately 26 studies conducted surveys and interviews to gain insight from study participants, as shown in the “Methods and Data” column. Some studies emphasized algorithms to analyze video, text, and sensor data. Overall, we observed that most studies evaluated AI from the user perspective and others leveraged AI to augment user performance.

**Table 1:** Evidentiary table of selected publications, summarizing their objectives, methods, participants, and outcomes (N=48).

Study	Objective	Methods and Data	Study participants	Immediate outcome observed
Aldape-Pérez et al [24]	To promote collaborative learning among less experienced physicians	Mathematical/ numerical data	NA^a^ (online database)	Delta Associative Memory was effective in pattern recognition in the medical field and helped physicians learn
Azari et al [25]	To predict surgical maneuvers from a continuous video record of surgical benchtop simulations	Mathematical/video data	37 surgeons	Machine learning’s prediction of surgical maneuvers was comparable to the prediction of robotic platforms
Balani and De Choudhury [26]	To detect levels of self-disclosure manifested in posts shared on different mental health forums on Reddit	Mathematical/text data	NA (Reddit posts from 7248 users)	Mental health subreddits can allow individuals to express or engage in greater self-disclosure
Cai et al [27]	To identify the needs of pathologists when searching for similar images, retrieved using a deep-learning algorithm	Survey study: Mayer’s trust model, NASA-TLX, questions for mental support for decision-making, diagnostic utility, workload, future use, and preference	12 pathologists	Users indicated having greater trust in SMILY; it offered better mental support, and providers were more likely to use it in clinical practice
Cvetković and Cvetković [28]	To analyze the influence of age, occupation, education, marital status, and economic condition on depression in breast cancer patients	Interview study using the Beck Depression Inventory guide	84 patients	Patient age and occupation had the most substantial influence on depression in breast cancer patients
Ding et al [29]	To learn about one’s health in everyday settings with the help of face-reading technology	Interview study: specific questions about time and location of usage, users’ perceptions and interpretations of the results, and intentions to use it in the future	10 users	Technology acceptance was hindered due to low technical literacy, low trust, lack of adaptability, infeasible advice, and usability issues
Erebak and Turgut [30]	To study human-robot interaction in elder care facilities	Survey study: Godspeed anthropomorphism scale, trust checklist [31], scales from [32], and automated functions of [33].	102 caregivers	No influence of anthropomorphism was detected on trust in robots; providers who trusted robots had more intention to work with them and preferred a higher automation level
Gao et al [34]	To detect motor impairment in Parkinson disease via implicitly sensing and analyzing users’ everyday interactions with their smartphones	Mathematical; sensor data	42 users	Parkinson disease was detected with significantly higher accuracy when compared to a clinical reference
Hawkins et al [35]	To measure the patient-perceived quality of care in US hospitals	Survey study; hospitals were asked to provide feedback regarding their use of Twitter for patient relations	NA (Tweets)	Patients use Twitter to provide input on the quality of hospital care they receive; almost half of the sentiment toward hospitals was, on average, favorable
Hu et al [36]	To detect lower back pain from body balance and sway performance	Mathematical; sensor data	44 patients and healthy participants	The machine-learning model was successful in identifying patients with back pain and responsible factors
Jin et al [37]	To identify, extract, and minimize medical error factors in the medication administration process	Mathematical/text data	NA (data from 4 hospitals)	The proposed machine-learning model identified 12 potential error factors
Kandaswamy et al [38]	To predict the accuracy of an order placed in the EHR^b^ by emergency medicine physicians	Mathematical/text and numerical data	53 clinicians	Machine-learning algorithms identified error rates in imaging, lab, and medication orders
Komogortsev and Holland [39]	To detect mild traumatic brain injury (mTBI) via the application of eye movement biometrics	Mathematical/video data	32 patients and healthy participants	Supervised and unsupervised machine learning classified participants with detection scores ≤ –0.870 and ≥0.79 as having mTBI, respectively
Krause et al [40]	To support the development of understandable predictive models	Mathematical/ numerical data	5 data scientists	Interactive visual analytic systems helped data scientists to interpret predictive models clinically
Ladstatter et al [41]	To measure the feasibility of artificial neural networks in analyzing nurses’ burnout process	Survey study: Nursing Burnout Scale Short Form	465 nurses	The artificial neural network identified personality factors as the reason for burnout in Chinese nurses
Ladstatter et al [42]	To assess whether artificial neural networks offer better predictive accuracy in identifying nursing burnouts than traditional statistical techniques	Survey study: Nursing Burnout Scale Short Form	462 nurses	Artificial neural networks identified a strong personality as one of the leading causes of nursing burnout; it produced a 15% better result than traditional statistical instruments
Lee et al [43]	To determine how wearable devices can help people manage their itching conditions	Interview study: user experience and acceptance of the device	40 patients and 2 dermatologists	Machine learning–based itchtector algorithm detected scratch movement more accurately when patients wore it for a longer duration
Marella et al [44]	To develop a semiautomated approach to screening cases that describe hazards associated with EHRs from a mandated, population-based reporting framework for patient safety	Mathematical/text and numerical data	NA	Naïve Bayes Kernel resulted in the highest classification accuracy; it identified a higher proportion of medication errors and a lower proportion of procedural error than manual screening
Mazilu et al [45]	To evaluate the impact of a wearable device on gait assist among patients with Parkinson disease	Interview study: asking about usability, feasibility, comfort, and willingness to use Gait Assist.	18 patients and 5 healthy participants	AI^c^-based Gait Assist was perceived as useful by the patients. Patients reported a reduction in freezing of gait duration and increased confidence during walking
McKnight [46]	To analyze patient safety reports.	Mathematical/text data	NA	Natural language processing improved the classification of safety reports as Fall and Assault; it also identified unlabeled reports
Moore et al [47]	To evaluate natural language processing’s performance for extracting abnormal results from free-text mammography and Pap smear reports.	Mathematical/text data	NA	The performance of natural language processing was comparable to a physician’s manual screening
Morrison et al [48]	To evaluate the usability and acceptability of ASSESS MS.	Interview study: feedback questionnaires, usability scales	51 patients, 6 neurologists, and 6 nurses	ASSESS MS was perceived as simple, understandable, effective, and efficient; both patients and doctors agreed to use it in the future
Muñoz et al [49]	To augment the relationship between physical therapists and their patients recovering from a knee injury, using a wearable sensing device	Interview study to understand how physical therapists work with their patients; user interface design considering usability and comfort	2 physical therapists	Machine learning–based wearable device correctly identified exercises such as leg lifts (100% accuracy) but also incorrectly identified three nonleg lifts as successfully performed leg lifts (3/18 false positives)
Nobles et al [50]	To identify periods of suicidality	Survey study: evaluating psychology students’ communication habits using electronic services	26 patients	The machine-learning model accurately identified 70% of suicidality when compared to the default accuracy (56%) of a classifier that predicts the most prevalent class
Ong et al [51]	To automatically categorize clinical incident reports	Mathematical/text and numerical data	NA	Naïve Bayes and support vector machine correctly identified handover and patient identification incidents with an accuracy of 86.29%-91.53% and 97.98%, respectively
Park et al [52]	To compare discussion topics in publicly accessible online mental health communities for anxiety, depression, and posttraumatic stress disorder	Mathematical/text data	NA	Depression clusters focused on self-expressed contextual aspects of depression, whereas the anxiety disorders and posttraumatic stress disorder clusters addressed more treatment- and medication-related issues
Patterson et al [53]	To understand how transparent complex algorithms can be used for predictions, particularly concerning imminent mortality in a hospital environment	Interview study: group discussion	3 researchers	All participants gave contradicting responses
Pryor et al [54]	To analyze the use of a software medical decision aid by physicians and nonphysicians	Observation study; the study indirectly tested the usability and users’ trust in the device	34 clinicians and 32 nonclinical individuals	Physicians did not follow tool recommendations, whereas nonphysicians used diagnostic support to make medical decisions
Putnam et al [55]	To describe a work-in-progress that involves therapists who use motion-based video games for brain injury rehabilitation	Interview study to understand therapists’ experiences, opinions, and expectations from motion-based gaming for brain injury rehabilitation	11 therapists and 34 patients	Identifying games that were a good match for the patient’s therapeutic objectives was important; traditional therapists’ goals were concentration, sequencing, coordination, agility, partially paralyzed limb utilization, reaction time, verbal reasoning, and turn-taking
Sbernini et al [56]	To track surgeons’ hand movements during simulated open surgery tasks and to evaluate their manual expertise	Mathematical/sensor data	18 surgeons	Strategies to reduce sensory glove complexity and increase its comfort did not affect system performance substantially
Shiner et al [57]	To identify inpatient progress notes describing falls	Mathematical/text data	NA	Natural language processing was highly specific (0.97) but had low sensitivity (0.44) in identifying fall risk compared to manual records review
Sonğur and Top [58]	To analyze clusters from 12 regions in Turkey in terms of medical imaging technologies’ capacity and use	Mathematical/text and numerical data	NA	The study identified inequities in medical imaging technologies according to regions in Turkey and hospital ownership
Swangnetr and Kaber [59]	To develop an efficient patient-emotional classification computational algorithm in interaction with nursing robots in medical care	Survey study: self-assessment manikin questionnaire to measure emotional response to the robot	24 residents	Wavelet-based denoising of galvanic skin response signals led to an increase in the percentage of correct classifications of emotional states, and more transparent relationships among physiological responses and arousal and valence
Wagland et al [60]	To analyze the patient experience of care and its effect on health-related quality of life	Survey study regarding treatment, disease status, physical activity, functional assessment of cancer therapy, and social difficulties inventory	NA	Nearly half of the total comments analyzed described positive care experiences. Most negative experiences concerned a lack of posttreatment care and insufficient information concerning self-management strategies or treatment side effects
Wang et al [61]	To evaluate a population health intervention to increase anticoagulation use in high-risk patients with atrial fibrillation	Mathematical/text and numerical data	NA (data from 14 primary care clinics)	After pharmacist review, only 17% of algorithm-identified patients were considered potentially undertreated
Waqar et al [62]	To analyze patients’ interest in selecting a doctor	Survey study: systems evaluation from patients’ and doctors’ perspectives	NA (data from 3 hospitals)	The proposed system solved the problem of doctor recommendations to a good effect when evaluated by domain experts
Xiao et al [63]	To achieve personalized identification of cruciate ligament and soft tissue insertions and, consequently, capture the relationship between the spatial arrangement of soft tissue insertions and patient-specific features extracted from the tibia outlines	Mathematical/image data	20 patients	The supervised learning and prediction method developed in this study provided accurate information on soft tissue insertion sites using the tibia outlines
Valik et al [64]	To develop and validate an automated Sepsis-3–based surveillance system in a nonintensive care unit	Mathematical/text and numerical data	NA	The Sepsis-3 clinical criteria determined by physician review were met in 343 of 1000 instances
Bailey et al [65]	To study the implementation of a clinical decision support system (CDSS) for acute kidney injury	Interview and observation study: organizational work of technology adoption	49 clinicians	Hospitals faced difficulties in translating the CDSS’s recommendations into routine proactive output
Carayon et al [66]	To improve the usability of a CDSS	Experimental study: simulation and observation to evaluate the usability	32 clinicians	Emergency physicians faced lower workload and higher satisfaction with the human factors–based CDSS compared to the traditional web-based CDSS
Parekh et al [67]	To develop and validate a risk prediction tool for medication-related harm in older adults	Mathematical/numerical data	1280 elderly patients	The tool used eight variables (age, gender, antiplatelet drug, sodium level, antidiabetic drug, past adverse drug reaction, number of medicines, living alone) to predict harm with a C-statistic of 0.69
Gilbank et al [68]	To understand the needs of the user and design requirements for a risk prediction tool	Survey and interview study: informal, semistructured meetings	15 stakeholders from hospitals, academia, industry, and nonprofit organizations	Nine physicians emphasized the need for a prerequisite for trusting the tool. Many participants preferred the technology to have roles complementary to their expertise rather than to perform tasks the physicians had been trained for. Having a tailored recommendation for a local context was deemed critical
Miller et al [69]	To understand the usability, acceptability, and utility of AI-based symptom assessment and advice technology	Survey study to measure ease of use	523 patients	425 patients reported that using the Ada symptom checker would not have made a difference in their care-seeking behavior. Most patients found the system easy to use and would recommend it to others
ter Stal et al [70]	To analyze the impact of an embodied conversational agent’s appearance on user perception	Interview study: Acosta and Ward Scale [71]	20 patients	The older male conversational agent was perceived as more authoritative than the young female agent (*P*=.03). Participants did not see an added value of the agent to the health app
Gabrielli et al [72]	To evaluate an online chatbot and promote the mental well-being of adolescents	Experimental, participatory design, and survey study to measure satisfaction	20 children	Sixteen children found the chatbot useful and 19 found it easy to use
Liang et al [73]	To develop a smartphone camera for self-diagnosing oral health	Interview Study to measure usability (NASA-TLX)	500 volunteers	Two experts agreed that OralCam could give acceptable results. The app also increased oral health knowledge among users
Chatterjee et al [74]	To access the feasibility of a mobile sensor-based system that can measure the severity of pulmonary obstruction	Mathematical/numerical data	91 patients, 40 healthy participants	Most patients liked using a smartphone as the assessment tool; they found it comfortable (mean rating 4.63 out of 5 with σ=0.73)
Beede et al [75]	To evaluate a deep learning–based eye-screening system from a human-centered perspective	Observation and interview study: unstructured	13 clinicians, 50 patients	Nurses faced challenges using the deep-learning system within clinical care as it would add to their workload. Low image quality and internet speed hindered the performance of the AI system

^a^NA: not applicable; these studies have only used data for their respective analyses without involving any human participant (user).

^b^EHR: electronic health record.

^c^AI: artificial intelligence.

We observed various algorithms in the final selection, with machine learning being the most common (n=18). Some studies also compared different algorithms based on analytical performance. However, few studies (n=5 against clinical/baseline standards, n=5 against clinicians) compared their AI models against a standard measure.

Table 2 summarizes the studies that used machine-learning algorithms. These studies emphasized algorithm development without considering human factors in substantial depth. In other words, the technological focus of many studies is currently on human-AI collaboration in health care while neglecting real-life clinical evaluation. Discussing studies that primarily focused on analytical performance is beyond the scope of this review. The general flaws and trends of such studies have been addressed in our prior work [7].

Overall, our review indicates that the dimensions of usability, user’s perception, workload, and trust in AI have been the most common interest of research in this field.

**Table 2:** Artificial intelligence (AI) studies that primarily focused on machine learning (ML) algorithm development (n=18).

Reference	AI/ML recommended by the study	Other AI/ML/non-AI used in the study	Proposed AI model(s) for comparison (1=compared; 0=not compared)			
			Other AI systems	Existing system (not AI)	Clinical or gold standard	Clinicians or user
Aldape-Pérez et al [24]	Delta Associative Memory	AdaBoostM1; bagging; Bayes Net; Dagging; decision table naïve approach; functional tree; logistic model trees; logistic regression; naïve Bayes; random committee; random forest random subspace; Gaussian radial basis function network; rotation forest; simple logistic; support vector machine	1	0	0	0
Azari et al [25]	Random forest and hidden Markov model	Not applicable	1	1	1	0
Balani and De Choudhury [26]	Perceptron	Naïve Bayes; k-nearest neighbor; decision tree	1	0	0	0
Cvetković and Cvetković [28]	Neural network and fuzzy logic	Not applicable	0	0	0	0
Gao et al [34]	AdaBoost	k-nearest neighbor, support vector machine, decision tree, random forest, naïve Bayes	1	1	1	0
Hu et al [36]	Deep neural network	Deep neural network with different inputs	1	0	0	0
Kandaswamy et al [38]	Random forest	Naïve Bayes; logistic regression; support vector machine	1	0	0	0
Komogortsev and Holland [39]	Supervised support vector machine	Unsupervised support vector machine and unsupervised heuristic algorithm developed by the authors	1	0	0	0
Marella et al [44]	Naïve Bayes kernel	Naïve Bayes; k-nearest neighbor; rule induction	1	0	0	1
Nobles et al [50]	Deep neural network	Support vector machine	1	0	0	0
Ong et al [51]	Naïve Bayes; support vector machine with radial-bias function	Support vector machine with a linear function	1	1	1	1
Shiner et al [57]	Natural language processing	Incident reporting system; manual record review	1	1	1	1
Wagland et al [60]	Did not recommend any particular algorithm	Support vector machine; random forest; decision trees; generalized linear models network; bagging; max-entropy; logi-boost	1	0	0	0
Waqar et al [62]	Hybrid algorithm developed by the authors	Not applicable	0	0	0	0
Xiao et al [63]	The authors developed a new algorithm	Linear regression with regularization; LASSO^a^; k-nearest neighbor; population mean	1	0	0	0
Valik et al [64]	The authors developed a new algorithm	Not applicable	0	0	1	1
Parekh et al [67]	The authors developed an algorithm based on multivariable logistic regression	Not applicable	0	1	0	0
Chatterjee et al [74]	Gradient boosted tree	Random forest, adaptive boosting	0	0	0	1

^a^LASSO: least absolute shrinkage and selection operator.

### Perception, Usability, Workload, and Trust

#### Perception

The perception of users was analyzed by several studies to adequately assess the quality of the proposed AI-based recommender system. Some studies incorporated perceptions of both patients and doctors [62,73] in developing their AI systems. Another study interviewed providers (therapists) about their experiences, opinions, expectations, and perceptions of a motion-based game for brain injury rehabilitation to guide the design of the proposed AI-based recommender system, which was a case-based reasoning (CBR) system [55]. The AI system ASSESS MS was also developed and evaluated based on users’ perceptions [48]. Studies included in our review that developed AI-based apps [27,29], AI robots [30], and wearable AI devices such as Gait Assist [45] and Itchtector [43] also accounted for users’ perceptions. From a psychological perspective, emotions might facilitate perception [76]. One study in our review measured users’ perception of an AI-based conversational agent [70], and another study developed an AI algorithm for real-time detection of patient emotional states and behavior adaptation to encourage positive health care experiences [59].

#### Usability

Some studies in our review performed usability testing of AI systems. For example, one study used AI to develop an adaptable CBR to help therapists ensure proper usability and functioning of CBR [55]. Guided by users’ needs, one study [27] developed an AI application (SMILY) to ensure good usability. Users found the clinical information to have higher diagnostic utility while using SMILY (mean 4.7) than while using the conventional interface (mean 3.7). They also experienced less effort (mean 2.8) and expressed higher trust (mean 6) in SMILY than with the conventional interface (mean 4.7; *P*=.01), as well as higher benevolence (mean 5.8 vs 2.6; *P*<.001). Another study included in our review noted the literacy gap as a significant hurdle in the usability of an AI-based face-reading app, and identified the impact of adaptability and cultural sensitivity as a limiting factor for usability [29]. Another study codesigned an AI chatbot with 20 students and performed a formative evaluation to better understand their experience of using the tool [72]. Two recent studies measured the perceived usability of AI-based decision-making tools: Ada, an AI tool that helps patients navigate the right type of care [69], and PE-Dx CDS, a tool for diagnosing pulmonary embolism [66]. However, in another study, the researchers primarily focused on developing the algorithm for assessing the severity of pulmonary obstruction and obtained users’ feedback on the end product [74]. Poor usability often leads to an increased workload, particularly when the user (provider or patient) is not trained in using the AI system, device, or app.

#### Workload

Caregivers are subject to workplace stress and cognitive workload, mostly due to the complexities and uncertainty of patient health and related treatment [77-79], and AI promises to minimize the health care workload through the automation of various levels. Nevertheless, if an AI system or program is poorly designed, the workload may possibly be elevated. Two studies in our review used a radial basis function network to assess burnout among nurses, and consequently captured the nonlinear relationship of the burnout process with the workload, work experience, conflictive interaction, role ambiguity, and other stressors [41,42]. The demand-control theory of work stress implies that workload abnormalities and job intensity can aggravate user fatigue by excessive workloads and trigger anxiety [80]. According to Maslach and Leiter [81], a mismatch between one’s skill sets (ability to perform a task) and responsibility (skills required to complete a task) intensifies users’ workload. Three studies in our review were invested in minimizing users’ workload by assessing the usability of AI systems such as ASSESS MS [48], Gait Assist [45], and SMILY [27].

#### Trust

Trust shapes clinicians’ and patients’ use, adoption, and acceptance of AI [6]. Trust is a psychological phenomenon that supports the inconsistency between the known (clinicians’ awareness, patient experience) and the unknown (deep-learning algorithms). Three studies included in our review measured user trust in health care AI systems. One study reported that the anthropomorphism of AI-based care robots has no influence on providers’ trust but was significantly related to the level of automation and intention to work with the robot [30]. This study proposed that providers who trusted robots more intended to work with them and preferred a higher automation level [30]. A recent perspective discusses the risk of overreliance or maximum trust in AI (automation) and instead suggests optimal trust between the user and AI system [6]. Besides experience, expertise, and prior knowledge, the performance of the AI technology also determines users’ trust. A study included in our review, using a poststudy questionnaire, found that doctors (pathologists) expressed higher trust in SMILY, an AI-based application, due to its better performance, interface, and higher benevolence compared with the conventional app [27]. By contrast, another study reported lower trust of experienced physicians in an AI-based recommendation tool due to its inefficient performance [54]. Based on patient data, expert physicians were able to identify the alternative and better explanation for patient health compared to the AI-based tool [54]. A recent study identified the impact of the AI interface on user’s trust [68]. Physicians in this study considered AI’s transparency and performance as facilitators of engendering trust.

### User-Centered Design

A user-centric design requires multidisciplinary cooperation between HFE experts, technologists, and end users. The inadequacy of a user-centered design also hinders user perception, usability, and trust, and increases the possibility of errors. The majority of the health care AI literature focuses on quantitative constraints, including performance metrics and precision, and is less focused on the user-centric development of AI technologies. Due to the lack of standard guidelines [7,16], not much research has invested in incorporating a user-centered design in AI-based technologies within the health care industry. In this review, we identified studies that performed experiments involving clinicians and patients, and consecutively evaluated their AI system’s (eg, app, wearable device) interface [27], applicability [27,29], and appearance (anthropomorphism) [30] to ensure user-centeredness. Other studies [43,45,48,49,55,62] also addressed user requirements such as wearability and privacy concerns. A recent study further acknowledged the importance of a user-centered clinical field study, and identified external factors such as low lighting, expensive image annotation, and internet speed that can deter the effectiveness of AI systems for diagnosing diabetic retinopathy [75].

## Discussion

### Main Findings

Research concerning AI in health care has shown promise for augmenting the quality of health care. However, there is a need for more theoretical advances and interventions that cover all levels and operations across the health care system. We need a systematic approach to safely and effectively bring AI into use, providing human factors, user-centered design, and delivery and implementation science. Many current AI models focus on engineering technology (informatics concepts) and do not sufficiently discuss the relevance of HFE in health care [82]. In this review, we explored and portrayed the involvement of HFE journals and conferences in health care AI research. We identified 48 studies, trending as more publications in recent years, which shows increased attention of the HFE community in this field.

Although advancement and focus have been made in the use of machine learning/AI to develop prediction and classification models, little research has been devoted to real-world translations with a user-centered design approach. To determine the diverse relationships between individuals and technology within a work environment, it is necessary to provide a better explanation as to how AI can be part of the overall health care system through a variety of HFE methods such as the Systems Engineering Initiative for Patient Safety (SEIPS) [83]. The SEIPS provides a framework that helps in comprehending the work system (people, tools and technologies, tasks, working environment, and organization), process (clinical process and process assisting the same), and outcomes (patient outcome, organizational outcome) in the health care domain [83]. This framework also helps to assess and understand the complex interaction between elements of the work system, and shows the impact of any technology-based intervention on the overall system [83].

This review also highlights the need for a systematic approach that evaluates AI’s impact (effectiveness) on patient care based on its computational capabilities and compatibility with clinical workflow and usability. Although some studies have acknowledged AI’s challenges from both humans factors (biases and usability) [84] and technical (quality of training data and standardization of AI) [7] standpoints, less emphasis has been given so far to the impact of AI integration into clinical processes [16] and services as well as to the user-centered design of AI systems for better human-AI interaction [84,85]. At this stage, where human beings and AI come together, challenges to human factors will likely arise.

### Next Steps

The next push for researchers should be to move AI research beyond solely model development into sociotechnical systems research and effectively use human factors principles. HFE researchers should consider users’ needs, capabilities, and interactions with other elements of the work system to ensure the positive impact of AI in transforming health care. Clinical systems are not inherently equivalent to predictable mechanical systems and need a systematic approach. One of the pivotal myths of automation is the assumption that AI can replace clinicians [33]. In fact, the use of AI can shape the activities and duties of clinicians, and might help them in their decision-making. In the domain of medical imaging, AI has shown great promise and is increasing rapidly. For instance, on January 18, 2021, an image analysis platform named AI Metrics received US Food and Drug Administration (FDA) 510(k) clearance [86]. Likewise, in the last 5 years, approximately 222 AI-based medical devices have been approved in the United States [87]. As AI continuous to grow, the associated risks also increase. Many health care AI systems are poorly designed and not evaluated thoroughly [14], and have neglected clinicians’ limited absorptive and cognitive capacities and their ability to use AI in clinical settings under a high cognitive workload [88-90]. Incorrect usage or misinterpretation of AI, similar to that of EHRs [91], may also result in patient harm. Therefore, more HFE research should focus on cognitive factors (biases, perceptions, trust), usability, situation awareness, and methodological aspects of AI systems.

### Usability

A user-centered design is essential for health care technologies, where the user is centrally involved in all phases of the design process [92]. However, when the user environment and activities are varied, designing standardized protocols for health care devices and software is complicated. As stated in this study, the problem further increases due to the heterogeneity of applications and AI variants. The human-computer interaction community has developed different user-centered design techniques. However, these methods are often underused by software development teams and organizations [93].

Usually, AI algorithms are complex, opaque, and thus difficult to understand. Therefore, it might be difficult for clinicians/end users to understand and interpret AI outcomes effectively without adequate instruction. Cognitive ergonomics is a fundamental principle dealing with usability issues [94]. Necessary procedural information stored in long-term memory is required to use a technical device [95]. Kieras and Polson [95] suggested the cognitive complexity theory (CCT) explicitly addressing the cognitive complexity of the user-to-device/interface interaction by explaining the user’s goals on the one hand and the computer system reaction on the other hand using production rules. The laws of production can be viewed as a series of rules in the form of IF conditions (display status) and THEN actions (input or action taken by the user). According to CCT, cognitive complexity is defined as the number of production rules segregated and learned in a specific action sequence. The definition of cognitive complexity in an AI-based health app can be as helpful as the definition of production rules (ie, the specification of what the system says and how users react) and factors that may contribute concurrently to the app’s complexity (ie, interface, menu structure, the language of communication, transparency of functions’ naming). It is, however, debatable whether the mere counting of production rules will reasonably assess the troubles perceived by users, considering that various factors contribute equitably to cognitive complexity. Cognitive computing systems [96], which are computing systems that can incorporate human-like cognitive abilities, can also augment and safeguard health care AI by making AI *adaptive* (learning from a changing environment, changing patient health, changing clinician’s requirements), interactive (easier human-AI interaction, better usability, easy to understand), iterative and stateful (narrowing down on the problem, considering past decisions/consequence while making current recommendations/tasks), and contextual (consider contextual elements) [96].

Moreover, challenges and hardships perceived by users might be a function of several factors not limited to the user’s experience, knowledge, intention of use, and working environment [97]. Therefore, an adaptable usability scale that encompasses the complexity of AI and the common usability factors applicable to that particular system or software should be created by HFE researchers. Perception of an AI system or its perceived ease of use can potentially be a function of users’ cognitive and physical abilities. Additionally, the obvious question is, where should user-centered design techniques and knowledge be considered in the life cycle of AI’s development?

### Trust and Biases

Human factors research on “automation surprises” primarily began with large-scale industrialization that involved autonomous technologies [84,98,99]. The automation surprise arises when an automated machine acts counterintuitively [100]. In health care, automation surprises might lead to confusion, higher workload, distrust, and inefficient operations [101]. In the health care environment, inadequate mental models and insufficient information about AI-based technology might lead to automation surprises and negatively influence trust [6]. Trust can also be hindered if an automated system tends to deter clinicians’ performance [6]. Research evaluating the performance of radiologists observed their deterring performance when aided by a decision support system [102]. Therefore, more HFE studies are needed that explore the factors and design requirements influencing users’ and clinicians’ optimal trust in AI. Future studies should also focus on patient trust in AI-generated recommendations.

When automated diagnostic systems are used in real-life clinics, they most likely are in the form of assistant or recommender systems where the AI system provides information to clinicians or patients as a second opinion. However, if the suggestions made by AI are entirely data-driven without accounting for the user’s opinion, as is the case for current designs, users could be biased toward or against the suggestion of the AI system [103]. Optimizing such user-AI trust interplay remains a challenge that HFE experts should consider as their future endeavor.

It should be noted that advocating for trust in automation for a prolonged time can also promote automation bias. Aviation studies have recorded instances of automation biases where pilots could not track vital flight indicators in the event of failure due to overreliance on the autopilot [104,105]. A review of automation bias focusing on the health care literature noted that the complexity of any assignment and the workload increased the likelihood of excessive reliance on automation [106], which can be detrimental to patient safety. Human factors such as cognitive ergonomics and a user-centered design should be utilized efficiently to minimize the health care AI system’s automation biases.

### Situation Awareness

Situation awareness is defined as “the perception of the elements in the environment within a volume of time and space, the comprehension of their meaning and the projection of their status in the near future” [107]. “Good” situation awareness is a prerequisite to better performance [84,107]. There might be an ongoing discussion around maximum versus optimum situation awareness. It is critical to understand that the optimum situation awareness is not necessarily the maximum situation awareness [108]. Maximizing the user’s situation awareness does not necessarily yield the best outcome (decisions from a human-AI collaboration) [108]. For example, concentrating on irrelevant details such as radio commercials, talking passengers, or the colors of other cars while driving may unnecessarily consume the driver’s working memory, increase the workload, or even act as a distraction [109].

Similarly, in a clinical setting, it is better to achieve optimal situation awareness rather than maximum situation awareness. Many studies have shown the deterring impact of excessive and unnecessary information on clinical work [110,111]. For example, false or irrelevant clinical alarms may increase the tension of nurses and even distract them. Performing critical health care tasks (such as administering narcotic medication, watching telemetry monitors) demands optimal situation awareness [112]; however, unnecessary or irrelevant situation awareness can disturb clinicians’ attention and working memory. Exploration of AI’s influence on clinicians’ situation awareness has not been studied extensively. More HFE-based research is needed to further explain the concept of optimal situation awareness in AI design. Both humans and AI each have skepticism regarding the information generated in their surroundings and extract the data that seem vital for clinical decision-making.

### Ecological Validation

The development, evaluation, and integration of sophisticated AI-based medical devices can be a challenging process requiring multidisciplinary engagement. It may enable a personalized approach to patient care through improved diagnosis and prognosis of individual responses to therapies, along with efficient comprehension of health databases. This solution has the power to reinvigorate clinical practices. Although the advent of personalized patient treatment is provocative, there is a need to evaluate the true potential of AI. The performance of AI depends on the quantity and quality of data available for training, as acknowledged in recent review papers [7,16]. Perhaps one of the most essential facts from the HFE viewpoint is that poor usability causes improper, inaccurate, and inefficient use [113]. Although the importance of usability testing and a user-centered design for medical devices has been substantially stated by the FDA [114] and other HFE experts, both regulatory guidelines and evaluation approaches fail to reflect the challenges faced by clinicians during their routine clinical activity [115]. In other words, most studies identified in our review were performed in a controlled environment, therefore lacking ecological validity. This finding is consistent with most other research in the field of AI and health care. Recent systematic reviews [7,16,116] analyzing AI’s role and performance in health care acknowledged that AI systems or models were often evaluated under unrealistic conditions that had minimal relevance to routine clinical practice.

Users under stress and discomfort might not be efficient in utilizing AI devices with poor usability. Unlike research or controlled settings, a clinical setting demands multitasking where clinicians (nurses) have to attend to several patients with different ailments. They also have to write clinical notes, monitor health fluctuations, administer critical medications, float to different departments during shortage of staff, educate new nurses, and respond to protocols in cases of emergency. Under such a working environment and cognitive workload, interpreting or learning to use an AI system that is not designed appropriately can be challenging and risky. Therefore, an AI system that perfectly qualifies usability tests in a research setting may fail in a clinical environment. Given these limitations, the few studies in our review that compared their AI model with clinical standards (see Table 2) are less relevant because the comparisons against clinical standards were made in an (ideal) controlled environment or without providing contextual information about the patient and the environment [117]. Moreover, the work system elements also differ substantially from an intensive care unit to an outpatient clinic. Therefore, AI-based medical systems must be evaluated in their respective clinical environment to ensure safer deployment.

### Limitations of the Review

This review does not include the complete available literature but was constrained within the selected journals and conferences. Studies investigating human-AI interactions in a health care context or leveraging HFE principles to evaluate health care AI systems published in non-HFE venues such as pure medical or informatics journals have not been included in this review. Notwithstanding these constraints, our analysis identified possible research gaps in the health disciplines that could, if addressed, help mobilize and integrate AI more efficiently and safely.

### Conclusion

HFE researchers should actively design and implement AI, and perform dynamical assessments of AI systems’ effects on interaction, workflow, and patient outcomes. An AI system is part of a greater sociotechnical system. Investigators with HFE expertise are essential when defining the dynamic interaction of AI within each element, process, and result of the work system. This means that we ought to adapt our strategy to the situations and contexts in the field; simultaneously, we must also find practical ways of generating more compelling evidence for our research.

## Appendix

Multimedia Appendix 1 Journal and conference proceedings list for the review.

## Abbreviations

AI: artificial intelligence
CBR: case-based reasoning
CCT: cognitive complexity theory
EHR: electronic health record
FDA: Food and Drug Administration
HFE: human factors and ergonomics
SEIPS: Systems Engineering Initiative for Patient Safety
