# Supplementary material for: Research Trends in Artificial Intelligence Applications in Human Factors Health Care: Mapping Review
Source: JMIR Hum Factors. 2021 Jun 18;8(2):e28236. doi: 10.2196/28236 (PMC8277302; doi:10.2196/28236)
Supplement: Multimedia Appendix 1 [file humanfactors_v8i2e28236_app1.docx]

# Appendices

- 1. Appendix A

| **Journal Name** | **SJR Quartile** | **SNIP** | **Country** |
| --- | --- | --- | --- |
| ACM Transactions on Computer-Human Interaction | Q2 | 1.823 | United States |
| Applied Ergonomics | Q1 | 1.903 | United States |
| Behavior and Information Technology | Q1 | 0.675 | England |
| BMJ Quality & Safety | Q1 | 3.243 | England |
| Cognition, Technology & Work | Q1 | 2.245 | United Kingdom |
| Computers in Human Behavior | Q1 | 3.079 | United States |
| Ergonomics | Q1 | 1.583 | United Kingdom |
| Ergonomics in Design | Q2 | 0.611 | United States |
| Foundations and Trends in  Human-Computer Interaction | Q2 | 2.837 | United States |
| Human-Computer Interaction | Q1 | 1.905 | United States |
| Human Factors | Q1 | 2.043 | United States |
| Human Factors and Ergonomics in Manufacturing | Q2 | 0.882 | United States |
| IEEE Transactions on Human-Machine Systems | Q1 | 2.168 | United States |
| IISE Transactions on Occupational Ergonomics and Human Factors | NA | NA | United States |
| International Journal of Human-Computer Studies | Q1 | NA0.688 | England |
| International Journal of Human-Computer Interaction | Q1 | 1.092 | United States |
| International Journal of Human Factors and Ergonomics | Q3 | 0.349 | United Kingdom |
| International Journal of Industrial Ergonomics | Q2 | 1.552 | Netherlands |
| Journal of Cognitive Engineering and Decision Making | Q1 | 1.611 | United States |
| Journal of Patient Safety | Q1 | 1.079 | United States |
| JMIR Human Factors | Q1 | NA | Canada |
| Theoretical Issues in Ergonomics | Q3 | 0.691 | United Kingdom |
| Nature Human Behavior | Q1 | 4.156 | United Kingdom |
| Accident Analysis & Prevention | Q1 | 2.296 | United Kingdom |

- 1. Appendix B

| **Conference Name** |
| --- |
| ACM Conference on Human-Computer Information Retrieval Symposium |
| Proceedings of the Human Factors and Ergonomics Society Annual Meeting |
| Asia Pacific Conference on Computer-Human Interaction |
| CHI Conference on Human Factors in Computing Systems |
| Proceedings on the International Symposium on Human Factors and Ergonomics in Health Care |
| IEEE International Workshop on Robot and Human Interactive Communication |
| Proceedings of ACM/IEEE International Conference on Human-Robot Interaction |
| Proceedings of the Australian Computer-Human Interaction Conference |
| Proceedings of the AHFE International Conference on Human Factors and Ergonomics in Healthcare and Medical Devices |
